# Extensive diversity of malaria parasites circulating in Central African bats and monkeys

**DOI:** 10.1002/ece3.4539

**Published:** 2018-10-05

**Authors:** Larson Boundenga, Barthélémy Ngoubangoye, Illich Manfred Mombo, Thierry Audrey Tsoubmou, François Renaud, Virginie Rougeron, Franck Prugnolle

**Affiliations:** ^1^ Centre International de Recherches Médicales de Franceville (CIRMF) Franceville Gabon; ^2^ Laboratoire MIVEGEC, UMR 224‐5290 CNRS‐IRD‐UM1‐UM2 Centre Hospitalier Régional Universitaire Montpellier France

**Keywords:** bats, diversity, gabon, *Hepatocystis*, monkeys, plasmodium

## Abstract

The order Haemosporidia gathers many protozoan parasites which are known to infect many host species and groups. Until recently, the studies on haemosporidian parasites primarily focused on the genus *Plasmodium* among a wide range of hosts. Genera, like the genus *Hepatocystis*, have received far less attention. In the present study, we present results of a survey of the diversity of *Hepatocystis* infecting bats and monkeys living in a same area in Gabon (Central Africa). Phylogenetic analyses revealed a large diversity of *Hepatocystis* lineages circulating among bats and monkeys, among which certain were previously observed in other African areas. Both groups of hosts harbor parasites belonging to distinct genetic clades and no transfers of parasites were observed between bats and monkeys. Finally, within each host group, no host specificity or geographical clustering was observed for the bat or the primate *Hepatocystis* lineages.

## 1 INTRODUCTION

The order of Haemosporidia gathers many protozoan parasites for which the most studied are the agents of human malaria. The haemosporidian parasites are known to infect many host species and groups, including birds, reptiles, and mammals (Boundenga, et al., 2017; Garnham, 1966). Currently, this order contains over 550 species that are classified in 17 extant genera (Martinsen & Perkins, 2013). However, the vast majority of described species have been assigned to the four genera *Plasmodium, Hepatocystis, Haemoproteus* and *Leucocytozoon* (Borner, et al., 2016; Boundenga, et al., 2016; Levine, 1980).

Up to now, the studies on haemosporidian parasites have primarily focused on the genus *Plasmodium* from a wide range of hosts. This interest in the genus *Plasmodium* was motivated by the desire to better understand the evolutionary history of human malaria agents and more particularly *Plasmodium falciparum* (the major causative agent of human malaria) (Perkins, 2014). Other genera like the genus *Hepatocystis* have historically received far less attention and only a couple of strains were genetically characterized up to recently where several studies, using molecular tools, have explored the diversity of *Hepatocystis* parasites infecting several groups of mammals (Ayouba, et al., 2012; Schaer, et al., 2013; Thurber, et al., 2013). One study performed in West Africa revealed that a large and yet unknown diversity of *Hepatocystis* parasites circulated in several species of bats (Schaer, et al., 2017). Others demonstrated a large diversity of this parasite genus in African primates (Ayouba, et al., 2012; Thurber, et al., 2013). Some bat, rodent, and monkey species in Asia were also shown to be infected with parasites of this genus (Hatta, Divis, & Singh, 2012; Olival, Stiner, & Perkins, 2007; Putaporntip, et al., 2010). Thus, all previous studies combined revealed that these parasites circulate in several mammal groups and that they are widely distributed (Ayouba, et al., 2012; Olival, et al., 2007; Putaporntip, et al., 2010; Schaer, et al., 2013, 2017 ). Nevertheless, all these studies dealt with the diversity in each group of hosts separately, without considering all hosts at the same time and the possibilities of transfers of these lineages among hosts.

In the present study, we analyzed the diversity of *Hepatocystis* parasites infecting different host groups (bats and monkeys) living in the same area. The study was performed at the primatology center of CIRMF (Centre International de Recherches Médicales de Franceville), which is located in the South‐East of Gabon and constitutes an interesting environment because different hosts (nonhuman primates and bats) dwell in a small area. Here, we wish to verify host specificity of *Hepatocystis* in monkeys and bats and evaluate if potential switches of *Hepatocystis* between these different mammal groups may occur.

## 2 MATERIALS AND METHODS

### 2.1 Sampling of animals’ blood

**Figure 1:**
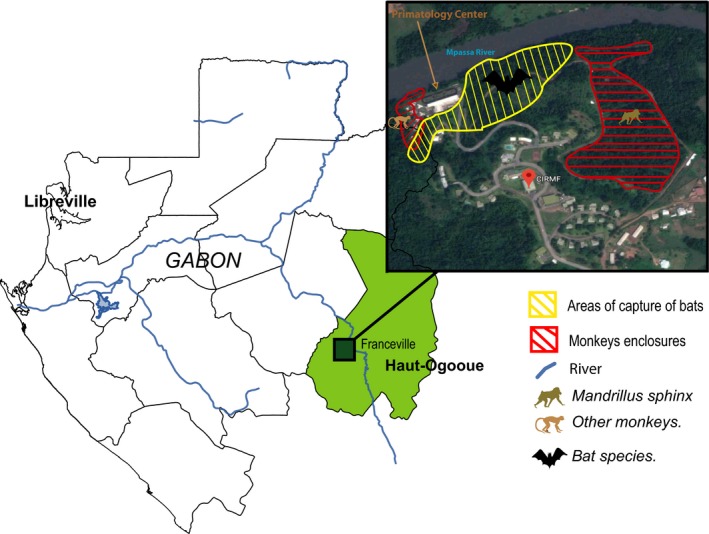
Study area. This map shows the location of Franceville in Gabon, the city in which the CIRMF is located. The box on the right is an aerial picture of the CIRMF in which are reported the areas of capture of bats (in yellow) and the enclosures in which the monkeys involved in the study live

Two hundred and sixty‐five blood samples were collected from animals living in and around the primatology center of CIRMF (1°37'59" N/13°34'59 "E), located in the city of Franceville in the province of Haut‐Ogooué, South‐East Gabon (Figure 1). The samples were taken on animals belonging to two groups: monkeys and bats. Regarding monkeys, animals live in semi‐free ranging enclosures for mandrills (250 individuals) and *Cercopithecus solatus* (13 members in this group). The other monkeys of this study (*Cercocebus torquatus*,* Cercopithecus cephus,* and *Cercopithecus nictitans*) live in different aviaries. Except for a few monkeys that were born in this center, all other nonhuman primates were born in the wild. For monkeys, 76 blood samples were collected during routine annual sanitary controls in 2014. The criteria of animal well‐being were guaranteed by the CIRMF veterinarians who proceeded to the blood sampling on the nonhuman primates. Two to seven millilitre of blood were collected within EDTA tube for each animal according to the weight. Tubes were then preserved in an icebox and transported to laboratories, where samples were conserved at −20°C.

The capture of bats occurred over a period of four months, between March and June 2014 (rainy season), using harp traps or mist nets (12 × 2.4 m) placed close to the area of storage of the food of primates just before twilight. Bats were captured at the proximity of the monkey enclosures because frugivorous bats tend to feed on food and bananas leftovers in the feeding areas of monkeys. Animals were identified by trained field biologists as previously described in (Maganga et al, 2014). Blood samples were collected from the brachial vein by puncture with a 0.4 × 13 mm gauge needle. The animals were subsequently released and marked with a microchip implant. All blood samples obtained were stored at −20°C. This study was done in agreement with the recommendations of the Gabonese National Ethics Committee (Authorization N°PROT/0020/2013I/SG/CNE). Animal handling by the CDP veterinarians complied with the criteria for animal care defined in the guidelines of the American Society of Mammalogists (http://www.mammalsociety.org/committees/animal-care-and-use).

### 2.2 Extraction of DNA, PCR and Phylogenetic analyses

All DNA samples were extracted from whole blood using the Qiagen DNeasy Blood & Tissue kit (Courteboeuf, France) according to manufacturer conditions and parasite infections were determined after amplification of a portion of parasite mitochondrial genome (cytochrome b: *cyt*‐b) as described in (Boundenga, et al., 2017; Prugnolle, et al., 2010). Around 10 µl of all PCR‐amplified products were send and used as templates for sequencing, after were confirmed as positive on 1.5% agarose gels in TAE buffer. This sequencing method did not allow us to highlight the co‐infections.

**Figure 2:**
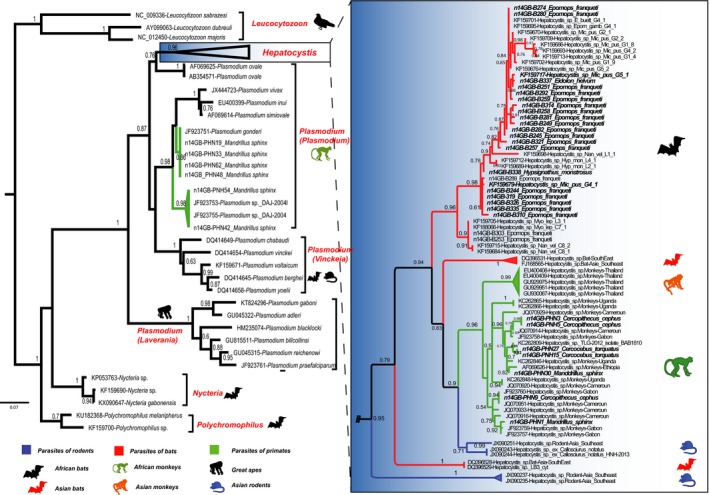
Phylogenetic relationships between the *cyt*‐b sequences of haemosporidian parasites obtained in our study (in bold) and the reference *cyt*‐b sequences obtained from existing databases and classified (with color codes and icons) according to their host species. The tree was constructed using a maximum likelihood method based on 696 bp‐long *cyt*‐b sequences. One thousand bootstrap replications were performed to assess confidence in topology. Branch colors indicate different groups of host species among vertebrates (red for bats, green for monkeys, blue for rodents and black for other parasites of mammals). For parasites of the genus *Plasmodium*, the subgenus is also provided (in brackets)

Phylogenetic analyses were performed using the *cyt*‐b sequences derived from chromatograms with no ambiguous base calls. To examine the relationship of the *cyt*‐b sequences obtained with the different haemosporidian species known so far, a phylogenetic tree was constructed using a set of reference sequences belonging to different species from Genbank (Supporting Information Table S1). Note that five of these sequences (the ones from Asian rodent *Hepatocystis*), although published by Genbank, have not been associated to any publication so far. Phylogenetic analyses were then performed after multiple alignments of the obtained partial *cyt*‐b sequences (696 nucleotides) and of the Genbank references sequences. We used both maximum likelihood (ML) (Figure 2 and Supporting Information Figure S1) and Bayesian (Supporting Information Figure S1) methods for tree construction. For the former, the best‐fitting model of sequence evolution based on the Akaike Information Criterion was GTR (General Time Reversible) + Gamma, as determined using ModelTest (Posada & Crandall, 1998). The highest‐likelihood DNA tree and the corresponding bootstrap support values were then obtained by using PhyML software (Dereeper, et al., 2008; Guindon, Lethiec, Duroux, & Gascuel, 2005) (freely available at the ATGC bioinformatics platform https://www.atgc-montpellier.fr/). maximum likelihood tree was rooted using *Leucocytozoon* species. For phylogenetic Bayesian inferences, the software MrBayes 3.2.6 under Geneious 11.0.5 was used (Huelsenbeck & Ronquist., 2001; Suchard et al., 2018). Bayesian posterior probabilities were computed under the same ML model by running two runs, four chains for 1,100,000 MCMC generations using the program default priors on model parameters. Mixing and convergence to stationary distributions were evaluated by inspecting graphically the trace of the log likelihood or sampled parameter values against the generation numbers. Trees were sampled from the posterior probability distribution every 200 generations and 100,000 trees were discarded as “burn‐in” to ensure that the chains have reached stationarity. A Majority‐rule consensus tree was retrieved from this study.

Finally, pairwise genetic distances (*p*‐distances) between different groups of sequences were estimated using MEGA 6.

## 3 RESULTS

A total of 265 samples were screened: 189 from four species of bats and 76 from five different species of NHPs (For more details see Table 1). Individuals from the two groups of vertebrates were found infected by haemosporidian parasites and belonging to different genera (*Hepatocystis* and *Plasmodium*). The infection rates are reported in Table 1.

**Table 1:** Description of the species of bats and monkeys analyzed in this study. The number of samples collected per host species, the prevalence of infection with a hemosporidian parasite in each host species and the parasite genus identified are indicated

Animal group	Family	Host species	Sample number	Prevalence %	Parasites genus
Bats	*Pteropodidae*	*Epomops franqueti*	160	13.12% (21/160)	*Hepatocystis*
	*Pteropodidae*	*Hypsignathus monstrosus*	21	4.8% (1/21)	*Hepatocystis*
	*Pteropodidae*	*Eidolon helvum*	6	16.67% (1/8)	*Hepatocystis*
	*Pteropodidae*	*Rousettus aegyptiacus*	2	0	*—*
Monkeys	*Cercopithecidae*	*Mandrillus sphinx*	50	16% (8/50)	*Plasmodium* and* Hepatocystis*
	*Cercopithecidae*	*Cercocebus torquatus*	5	40% (2/5)	*Hepatocystis*
	*Cercopithecidae*	*Cercopithecus solatus*	13	**0**	*—*
	*Cercopithecidae*	*Cercopithecus cephus*	4	75% (3/4)	*Hepatocystis*
	*Cercopithecidae*	*Cercopithecus nictitans*	4	0	*—*

**Figure 3:**
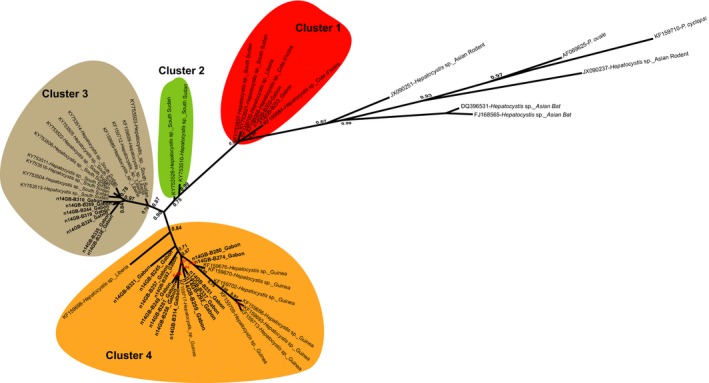
Phylogenetic relationships between the *cyt‐b* sequences of *Hepatocystis* of African bats with some outgroups (e.g. Hepatocystis of Asian bats, *P. ovale*…). The unrooted tree was inferred from 696 bp nucleotides. The tree was performed using a maximum likelihood method. One thousand bootstrap replications were performed to assess confidence in topology (only values above 80% are shown). Four genetic clusters supported by high bootstrap values are highlighted with colors. *Cyt*‐b sequences obtained in our study are in bold

**Figure 4:**
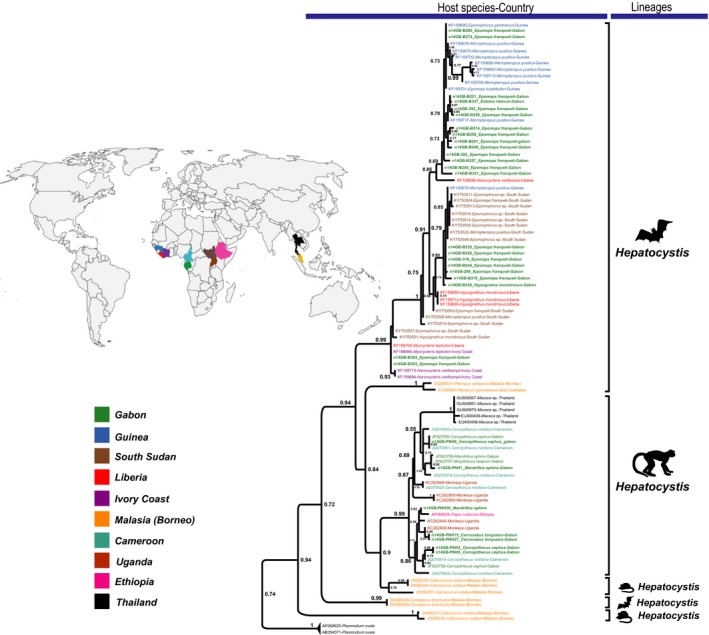
Phylogeny of the *cyt*‐b sequences of haemosporidian parasites obtained in our study and from other studies and classified (with color codes) according to their geographical origin (map performed using Adobe illustrator CS6 software). *Hepatocystis* sequences are identified with a color code by country. At the bottom of the tree is represented the country in which the different bats were captured

Regarding bats, four species were collected inside the CIRMF: *Epomops franqueti*,* Hypsignathus monstrosus, Eidolon helvum,* and* Rousettus aegyptiacus*. We found 12.2% of individuals sampled infected by haemosporidian parasites, belonging to three of the four bats species sampled. The infections rates varied from one species to another: *E. franqueti* 13.12% (21/160), *H. monstrosus* 4.8% (1/21) and *E. helvum* 16.67% (1/8) (Table 1). Phylogenetic analyses revealed that haemosporidian parasites identified among the bats species clustered with several lineages of the *Hepatocystis* genus identified previously in other African areas (Figure 2 and Supporting Information Figure S1). Thus, all lineages obtained in this study infecting African bats belonged to a monophyletic group including all lineages recently described in several species of West Central and East African bats (Figure 2). In our phylogeny, we can discriminate at least four distinct clades infecting the African bats (clade 1–4 in Figure 3). These clades are well supported (high bootstrap values) and the average divergences (*p‐*distance) measured between each pair of clade vary from 0.02 to 0.03, a range similar to the divergence measured between *P. falciparum* and *P. reichenowi* (0.03). As previously noted by Schaer et al. (2013), this observation suggests that the group of African *Hepatocystis* from bats actually comprises different *Hepatocystis* species. Phylogenetic analyses revealed that all sequences of parasites identified during our study and all different lineages from diverse geographical zones (countries) are mixed throughout the tree for African chiropterans. Thus, analyses indicate no strict geographic distribution within *Hepatocystis* isolates identified (Figure 4).

**Figure 5:**
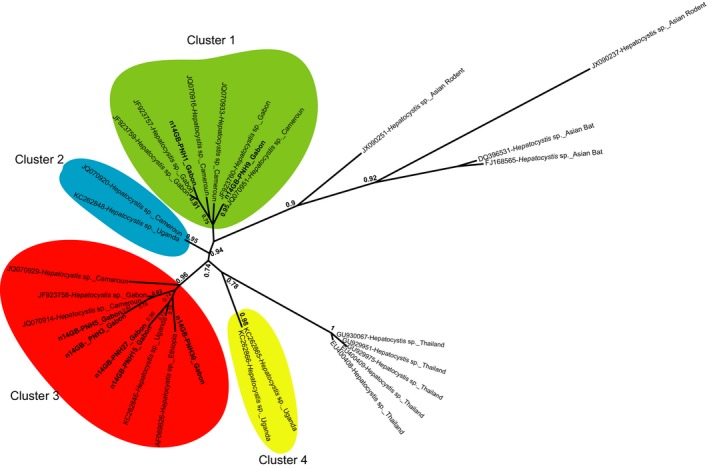
Phylogenetic relationships between the *Cyt‐b* sequences of *Hepatocystis* of African monkeys with some outgroups (e.g. *Hepatocystis* of Asian bats and rodents…). The unrooted tree was inferred from 696 bp nucleotides. The tree was performed using a maximum likelihood method. One thousand bootstrap replications were performed to assess the confidence in topology (only values above 80% are shown). Four genetic clusters supported by high bootstrap values are highlighted with colors. *Cyt*‐b sequences obtained in our study are in bold

Beside the chiropteran hosts, five monkey species were included in the study: *Mandrillus sphinx, Cercocebus torquatus, Cercophithecus cephus, Cercopithecus nictitans,* and *Cercophithecus solatus*. Three of them (*M. sphinx* 16% [8/50], *C. torquatus* 40% [2/5] and *C. cephus* 75% [3/4]) were found infected with haemosporidian parasites (representing nearly 22.03% of individuals infected) belonging either to the *Hepatocystis* or the *Plasmodium* genus (Table 1). Regarding the *Hepatocystis*, they form a monophyletic group. This group comprises parasites from both African and Asian monkeys and is closely related to the *Hepatocystis* of Asian rodents and bats (Figure 2 and Supporting Information Figure S1). Among the African primates, phylogenetic analyses suggest the existence of at least four well‐supported clades (Figure 5) which average divergence (*p‐*distance) range from 0.02 to 0.04, again estimates close to the divergence estimated between well‐defined species (e.g., 0.03 between *P. falciparum* and *P. reichenowi*). Within the tree, we observe no grouping of sequences by geographical areas, rather a mixed of sequences from different countries and no grouping by host species or genus (Figure 4). No host switch was observed between bats and primates (i.e., no primate *Hepatocystis* were found among bats or vice versa). For the *Plasmodium* species infected monkeys, we identified two parasites: *Plasmodium* sp.‐DAJ‐2004 and *Plasmodium gonderi*. These species were only found among mandrills.

## 4 DISCUSSION

The discovery of new species in groups of parasites yet historically well studied because of their proximity to human malaria agents (e.g., the primate malaria) has revived the interest to explore the diversity of extant malaria parasites in different host groups using molecular methods (Boundenga et al., 2015; Megali, Yannic, & Christe, 2011; Schaer, et al., 2015). In this study, we investigated the diversity of extant malaria parasites circulating among bats and monkeys living in the same area, using molecular tools to analyze the parasite content of each host species collected and determine whether transfers of parasites may occur among host groups.

Molecular analyses revealed that the individuals of different groups of mammals (primates and bats) are infected by haemosporidian parasites belonging to at least two distinct genera: *Plasmodium* and* Hepatocystis* (the focus of this study) (Figure 2 and Table 1). No parasites of *Polychromophilus* and *Nycteria* genera which were previously reported among bats (Duval, et al., 2012; Karadjian, et al., 2016) were found during this study.

Regarding the genus *Hepatocystis*, they were found in bats and primates in our study. In the Gabonese bats, we observed a large diversity of lineages belonging to distinct clades. Most of these lineages were genetically close or similar to lineages recently described in West and East African bats (Schaer, et al., 2013, 2017 ). Such diversity of sequences may reflect the presence of different species of *Hepatocystis* or a single species with a large geographical distribution and a complex evolutionary history that led to the maintenance of divergent lineages within this species. The divergences observed between clades (which are close or higher than the divergences measured between well‐described species of *Plasmodium*) are rather in favor of the presence of distinct species.

As previously noted by Schaer et al. (2017), no clear geographic patterns nor grouping according to the bat species or the bat genus could be observed in the distribution of these lineages in the phylogeny. They generally display a large geographical range but also a large host range, infecting different species and even different genera (Figure 4). Thus, certain lineages observed in *Epomops franqueti* from our study area in Gabon were also described from the same host species in Uganda or South Sudan or from a different bat genus (e.g., *Micropteropus pusillus*). This lack of clustering according to host and geography may be of multiple origins and future studies exploring this in more details should be done. This will necessitate more systematic data on the diversity of *Hepatocystis* in the African bats all over the continent and in more host species. This variability could, for instance, be related to the historical expansion and contraction of bat distribution ranges and their secondary or primary contacts.

The diversity of lineages observed in bats varies from one species to another. In our study, most lineages were for instance observed from only one species of bats, *E. franqueti*. This could obviously be linked to sample size that was far higher for this species than for the others but it has been demonstrated for other pathogen groups like viruses that ecological factors such as the home range, the size of the animals, the roosting patterns, or the physiological characteristics may also explain these variations in the diversity of pathogen communities among species (Maganga, et al., 2014). Again more systematic data on the diversity of *Hepatocystis* in bats will be needed to explore such aspects in details.

To our knowledge, our study is the first to report infections with haemosporidian parasites in *Eidolon helvum*. This result needs nevertheless to be considered with caution. PCR methods are known to be very sensitive and may, in certain cases, detect DNA of parasites in the blood of a wrong host after the parasites have died (Vafa Homann et al., 2017). The only valid proof of an infection would be the observation of the parasites in a blood smear or, at least, if only molecular analyses are available, confirmation of infection in multiple individuals of the same species.

Regarding the *Hepatocystis* found in the African monkeys, they form a monophyletic clade with the *Hepatocystis* from the Asian monkeys, distinct from that of the African bats (Putaporntip, et al., 2010; Seethamchai, Putaporntip, Malaivijitnond, Cui, & Jongwutiwes, 2008). The entire clade is a sister group of the clade formed by the *Hepatocystis* of Asian bats and rodents (Cox‐Singh et al., 2006) (Figure 2). As in bats, the *Hepatocystis* from African monkeys form a diverse clade and it is yet unclear whether these *Hepatocystis* lineages are the image of multiple taxa or a single widely dispersed species (Ayouba, et al., 2012). Nevertheless, as previously noted for the bat *Hepatocystis*, the level of divergence observed between clades is of similar order of magnitude as the one observed between well‐defined species of *Plasmodium* parasites. So it is likely that these different lineages correspond to different species. Our study and previous ones (Ayouba, et al., 2012; Prugnolle, et al., 2011; Thurber, et al., 2013) suggest that several African monkey species can be infected with these different parasite lineages, which do not seem to cluster according to host species or genus.

No transfers of *Hepatocystis* have been observed in our study between bats and primates despite the fact that they were captured in the same area and that all hosts share the same habitat. This observation is somehow in contrast with the history of this genus that was marked by multiple and recurrent host switches between bats, rodents, and primates. Several explanations may be given to explain the absence of transfers between sympatric populations of hosts: (a) the parasites of each host species do not encounter the other host species or (b) there is a strong host specificity that prevent the transfers. Regarding the first hypothesis, this could be linked to the absence of vectors playing the role of bridge between the different host species. Very few are known regarding the vectors of *Hepatocystis* except that they might be *Culicoides* (Thurber, et al., 2013). For the second hypothesis, nothing is known about the factors involved in the invasion of the host by species of this genus and this again should be an area of future researches.

From an evolutionary point of view, the sequences of *Hepatocystis* available so far suggest that the history of this group of parasites has been complex, characterized by repeated host shifts between different host groups associated to repeated events of geographic expansion from Africa to Asia and *vice versa*. Nevertheless, data are still too scarce to draw robust scenarios to explain the observed relationships between the parasites of different host and geographic areas. A more systematic analysis of the diversity of these parasites in a wider range of hosts covering larger geographic areas would be needed to understand this history. The analysis of the genetic relationships between these different hosts would certainly also be a plus. However, parasites of this lineage are genetically closely related and completely included in the group of parasites belonging to the genus *Plasmodium* (Supporting Information Figure S2). Thus, as previously suggested, we think that a complete revision of this genus should be performed in light of these recent results.

To finish, a couple of words regarding the *Plasmodium* species found in monkeys. They belonged to two species. *P. gonderi* and *P. sp._*DAJ‐2004. If *P. gonderi* is well known and well described for a long time, *P. sp._*DAJ‐2004 was only molecularly described for the first time in 2004, isolated from a drill (*Manrdrillus leucopheaus*). Since then, infections were observed mainly in mandrills (*Mandrillus sphinx*) and in only a few occasions (in particular unnatural settings) in some *Cercopithecus* species (Faust & Dobson, 2015; Prugnolle, et al., 2011). Until a formal description of this new species is written, we now propose to name this species in reference to the genus of primates (genus *Mandrillus*) that seems to represent its principal host: *Plasmodium mandrilli*. Although we acknowledge that naming this species does not follow the taxonomical standards, we nevertheless think that providing a name for this lineage should ease future discussion on it.

## CONFLICT OF INTEREST

The authors declare that they have no competing interests.

## AUTHORS’ CONTRIBUTION

LB, BN, and FP designed this study; LB, BN, TAT and IMM contributed to the acquisition of samples in fieldwork; LB, FP, VR, IMM, and FP contributed to data interpretation, FP, FR and LB conducted and supervised this work, LB wrote manuscript, FP, VR and FR, revised. All authors read and approved the final manuscript.

## DATA ACCESSIBILITY

All sequences obtained (accession numbers: MG602631–MG602666) and used are accessible in GenBank.
