# Supplementary material for: Extensive diversity of malaria parasites circulating in Central African bats and monkeys
Source: Ecol Evol. 2018 Oct 5;8(21):10578–86. doi: 10.1002/ece3.4539 (PMC6238140; doi:10.1002/ece3.4539)
Supplement: Supplementary file 1 [file ECE3-8-10578-s001.pdf]

# Supplementary data

## Legends

**Figure S1.** Phylogeny tree for Hepatocystis parasites recovered by Bayesian analysis of cytochrome-b gene. This tree was built with Bayesian method using partial *cytochrome b* sequences (see Material and Methods for details regarding the cyt-b sequence used)

**Figure S2.** Simple phylogeny of malaria parasites highlighting the position of the genus *Hepatocystis i*nside the *Plasmodium* genus. This tree was built with maximum likelihood method using partial *cytochrome b* sequences (see Material and Methods for details regarding the cyt-b sequence used).

**Table S1**. List of all sequences used in this study, including the sequences from Genbank and the ones obtained in our study.

# Figure S1

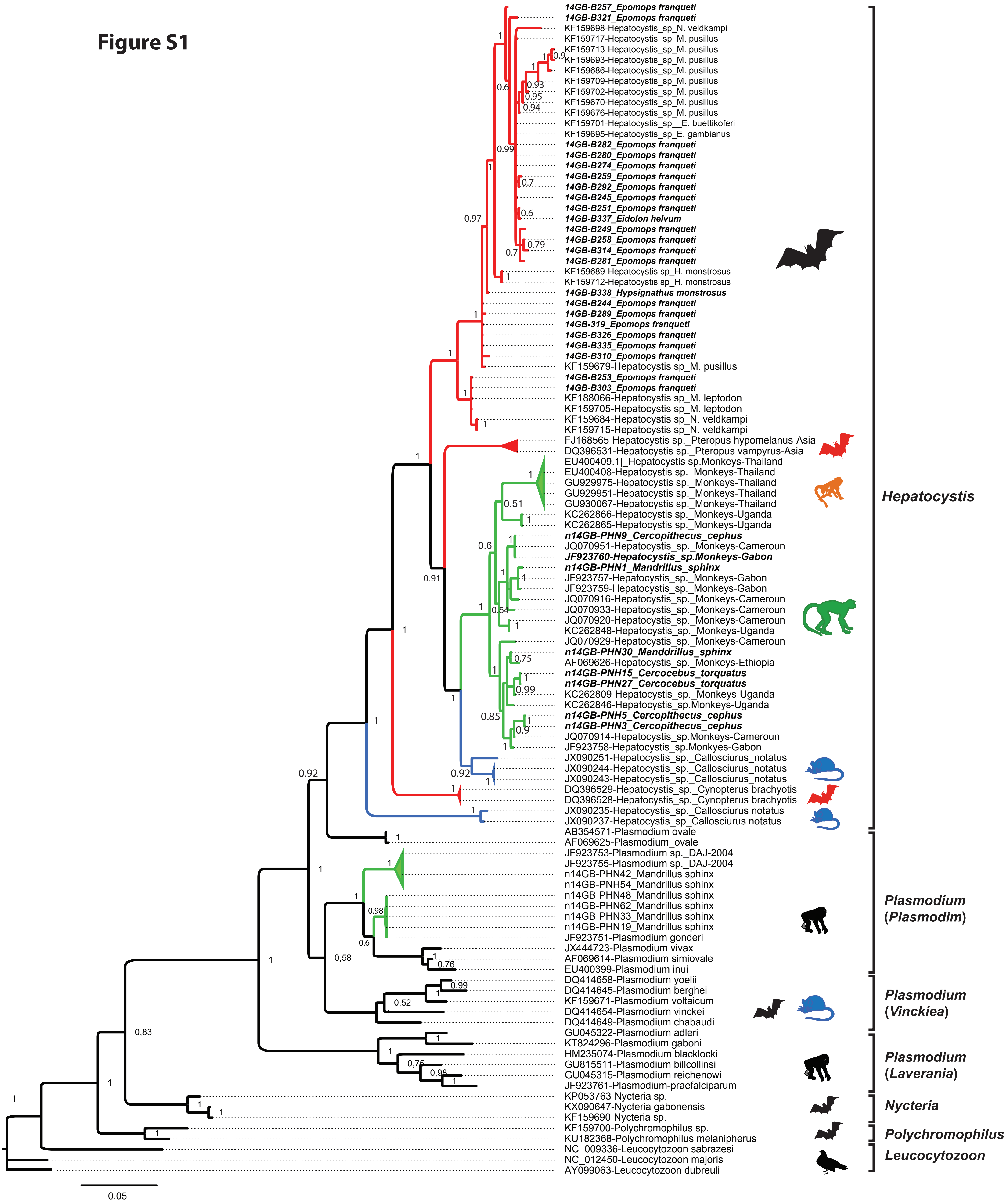

14GB-B257_Epomops franqueti
14GB-B321_Epomops franqueti
KF159698-Hepatocystis_sp_N. veldkampi
KF159717-Hepatocystis_sp_M. pusillus
KF159713-Hepatocystis_sp_M. pusillus
KF159693-Hepatocystis_sp_M. pusillus
KF159686-Hepatocystis_sp_M. pusillus
KF159709-Hepatocystis_sp_M. pusillus
KF159702-Hepatocystis_sp_M. pusillus
KF159670-Hepatocystis_sp_M. pusillus
KF159676-Hepatocystis_sp_M. pusillus
KF159701-Hepatocystis_sp__E. buettikoferi
KF159695-Hepatocystis_sp_E. gambianus
14GB-B282_Epomops franqueti
14GB-B280_Epomops franqueti
14GB-B274_Epomops franqueti
14GB-B259_Epomops franqueti
14GB-B292_Epomops franqueti
14GB-B245_Epomops franqueti
14GB-B251_Epomops franqueti
14GB-B337_Eidolon helvum
14GB-B249_Epomops franqueti
14GB-B258_Epomops franqueti
14GB-B314_Epomops franqueti
14GB-B281_Epomops franqueti
KF159689-Hepatocystis sp_H. monstrosus
KF159712-Hepatocystis sp_H. monstrosus
14GB-B338_Hypsignathus monstrosus
14GB-B244_Epomops franqueti
14GB-B289_Epomops franqueti
14GB-319_Epomops franqueti
14GB-B326_Epomops franqueti
14GB-B335_Epomops franqueti
14GB-B310_Epomops franqueti
KF159679-Hepatocystis sp_M. pusillus
14GB-B253_Epomops franqueti
14GB-B303_Epomops franqueti
KF188066-Hepatocystis sp_M. leptodon
KF159705-Hepatocystis sp_M. leptodon
KF159684-Hepatocystis sp_N. veldkampi
KF159715-Hepatocystis sp_N. veldkampi
FJ168565-Hepatocystis sp._Pteropus hypomelanus-Asia
DQ396531-Hepatocystis sp._Pteropus vampyrus-Asia
EU400409.1|_Hepatocystis sp.Monkeys-Thailand
EU400408-Hepatocystis sp._Monkeys-Thailand
GU929975-Hepatocystis sp._Monkeys-Thailand
GU929951-Hepatocystis sp._Monkeys-Thailand
GU930067-Hepatocystis sp._Monkeys-Thailand
KC262866-Hepatocystis sp._Monkeys-Uganda
KC262865-Hepatocystis sp._Monkeys-Uganda
n14GB-PHN9_Cercopithecus_cephus
JQ070951-Hepatocystis_sp._Monkeys-Cameroun
JF923760-Hepatocystis_sp.Monkeys-Gabon
n14GB-PHN1_Mandrillus_sphinx
JF923757-Hepatocystis_sp._Monkeys-Gabon
JF923759-Hepatocystis_sp._Monkeys-Gabon
JQ070916-Hepatocystis_sp._Monkeys-Cameroun
JQ070933-Hepatocystis_sp._Monkeys-Cameroun
JQ070920-Hepatocystis_sp._Monkeys-Cameroun
KC262848-Hepatocystis_sp._Monkeys-Uganda
JQ070929-Hepatocystis_sp._Monkeys-Cameroun
n14GB-PHN30_Manddrillus_sphinx
AF069626-Hepatocystis_sp._Monkeys-Ethiopia
n14GB-PNH15_Cercocebus_torquatus
n14GB-PHN27_Cercocebus_torquatus
KC262809-Hepatocystis_sp._Monkeys-Uganda
KC262846-Hepatocystis_sp.Monkeys-Uganda
n14GB-PNH5_Cercopithecus_cephus
n14GB-PHN3_Cercopithecus_cephus
JQ070914-Hepatocystis_sp.Monkeys-Cameroun
JF923758-Hepatocystis_sp.Monkyes-Gabon
JX090251-Hepatocystis_sp._Callosciurus_notatus
JX090244-Hepatocystis_sp._Callosciurus_notatus
JX090243-Hepatocystis_sp._Callosciurus_notatus
DQ396529-Hepatocystis_sp._Cynopterus brachyotis
DQ396528-Hepatocystis_sp._Cynopterus brachyotis
JX090235-Hepatocystis_sp._Callosciurus_notatus
JX090237-Hepatocystis_sp_Callosciurus notatus
AB354571-Plasmodium ovale
AF069625-Plasmodium_ovale
JF923753-Plasmodium sp._DAJ-2004
JF923755-Plasmodium sp._DAJ-2004
n14GB-PHN42_Mandrillus sphinx
n14GB-PNH54_Mandrillus sphinx
n14GB-PHN48_Mandrillus sphinx
n14GB-PHN62_Mandrillus sphinx
n14GB-PHN33_Mandrillus sphinx
n14GB-PHN19_Mandrillus sphinx
JF923751-Plasmodium gonderi
JX444723-Plasmodium vivax
AF069614-Plasmodium simiovale
EU400399-Plasmodium inui
DQ414658-Plasmodium yoelii
DQ414645-Plasmodium berghei
KF159671-Plasmodium voltaicum
DQ414654-Plasmodium vinckei
DQ414649-Plasmodium chabaudi
GU045322-Plasmodium adleri
KT824296-Plasmodium gaboni
HM235074-Plasmodium blacklocki
GU815511-Plasmodium billcollinsi
GU045315-Plasmodium reichenowi
JF923761-Plasmodium-praefalciparum
KP053763-Nycteria sp.
KX090647-Nycteria gabonensis
KF159690-Nycteria sp.
KF159700-Polychromophilus sp.
KU182368-Polychromophilus melanipherus
NC_009336-Leucocytozoon sabrazesi
NC_012450-Leucocytozoon majoris
AY099063-Leucocytozoon dubreuli
Hepatocystis
Plasmodium (Plasmodim)
Plasmodium (Vinckiea)
Plasmodium (Laverania)
Nycteria
Polychromophilus
Leucocytozoon
0.05

Figure S2

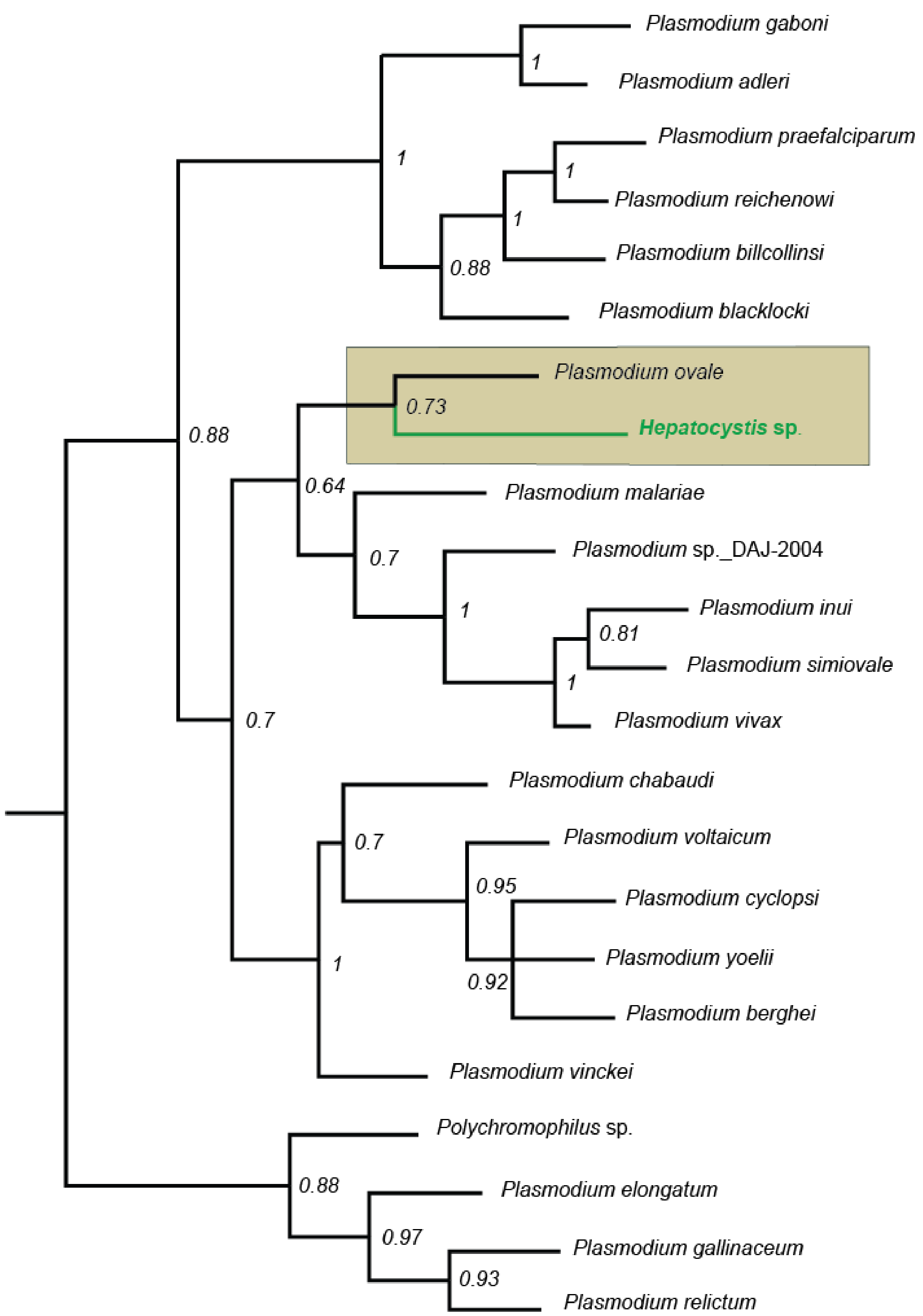
Plasmodium gaboni
1
Plasmodium adleri
Plasmodium praefalciparum
1
1
Plasmodium reichenowi
1
Plasmodium billcollinsi
0.88
Plasmodium blacklocki
Plasmodium ovale
0.73
Hepatocystis sp.
0.88
0.64
Plasmodium malariae
0.7
Plasmodium sp._DAJ-2004
1
Plasmodium inui
0.81
Plasmodium simiovale
1
0.7
Plasmodium vivax
Plasmodium chabaudi
0.7
Plasmodium voltaicum
0.95
Plasmodium cyclopsi
Plasmodium yoelii
1
0.92
Plasmodium berghei
Plasmodium vinckei
Polychromophilus sp.
0.88
Plasmodium elongatum
0.97
Plasmodium gallinaceum
0.93
Plasmodium relictum

## Table S1

| Lineage | Accession number of sequence (cytb) | Host | Isolates | Group | country |
|---|---|---|---|---|---|
| *Hepatocystis* sp. | KF159695 | *Epomophorus gambianus* | Epom_gamb_G4_1 | Bat | Guinea |
| | KF159701 | *Epomops buettikoferi* | E_buett_G4_1 | Bat | *Guinea* |
| | KF159676 | *Micropteropus pusillus* | Mic_pus_G5_2 | Bat | Guinea |
| | KF159670 | *Micropteropus pusillus* | Mic_pus_G2_1 | Bat | Guinea |
| | KF159702 | *Micropteropus pusillus* | Mic_pus_G1_9 | Bat | Guinea |
| | KF159686 | *Micropteropus pusillus* | Mic_pus_G1_8 | Bat | Guinea |
| | KF159693 | *Micropteropus pusillus* | Mic_pus_G4_2 | Bat | Guinea |
| | KF159713 | *Micropteropus pusillus* | Mic_pus_G1_4 | Bat | Guinea |
| | KF159709 | *Micropteropus pusillus* | Mic_pus_G2_2 | Bat | Guinea |
| | KF159717 | *Micropteropus pusillus* | Mic_pus_G5_1 | Bat | Guinea |
| | KF159679 | *Micropteropus pusillus* | Mic_pus_G4_1 | Bat | Guinea |
| | KF159698 | *Nanonycteris veldkampii* | Nan_vel_L1_1 | Bat | Liberia |
| | KF159689 | *Hypsignathus monstrosus* | Hyp_mon_L2_1 | Bat | Liberia |
| | KF159712 | *Hypsignathus monstrosus* | Hyp_mon_L4_1 | Bat | Liberia |
| | KF159689 | *Hypsignathus monstrosus* | Hyp_mon_L2_1 | Bat | Liberia |
| | KF159705 | *Myonycteris leptodon* | Myo_lep_L3_1 | Bat | Liberia |
| | KF159684 | *Nanonycteris veldkampii* | Nan_vel_C8_1 | Bat | Ivory Coast |
| | KF159715 | *Nanonycteris veldkampii* | Nan_vel_C8_2 | Bat | Ivory Coast |
| | KF188066 | *Myonycteris leptodon* | Myo_lep_C7_1 | Bat | Ivory Coast |
| | KY753521 | *Hypsignathus monstrosus* | Hyps_DMR553 | Bat | South Sudan |
| | KY753507 | *Epomophorus sp.* | Epom_DMR299 | Bat | South Sudan |
| | KY753510 | *Epomophorus sp.* | Epom_DMR632 | Bat | South Sudan |
| | KY753526 | *Micropteropus pusillus* | Mic_DMR546 | Bat | South Sudan |
| | KY753503 | *Epomops franqueti* | Epo_DMR592 | Bat | South Sudan |
| | KY753506 | *Epomophorus sp.* | Epom_DMR162 | Bat | South Sudan |
| | KY753522 | *Micropteropus pusillus* | Mic_DMR134 | Bat | South Sudan |
| | KY753505 | *Epomophorus* sp. | Epom_DMR161 | Bat | South Sudan |

| | | | | | |
|---|---|---|---|---|---|
| *Hepatocystis* sp. | KY753514 | *Epomophorus* sp. | Epom_DMR738 | Bat | South Sudan |
| | KY753516 | *Epomophorus* sp. | Epom_DMR862 | Bat | South Sudan |
| | KY753513 | *Epomophorus* sp. | Epom_DMR735 | Bat | South Sudan |
| | KY753504 | *Epomops franqueti* | Epo_DMR595 | Bat | South Sudan |
| | KY753511 | *Epomophorus* sp. | Epom_DMR727 | Bat | South Sudan |
| | DQ396531 | *Pteropus vampyrus* | PP1 | Bat | Malasia |
| | FJ168565 | *Pteropus hypomelanus* | - | Bat | Asie Southeast |
| | DQ396528 | *Cynopterus brachyotis* | IZ09 | Bat | Malasia |
| | DQ396529 | *Cynopterus brachyotis* | LB3 | Bat | Malasia |
| | **MG602650** | *Hypsignatus monstrosus* | n14GB-B338 | Bat | Gabon |
| | **MG602649** | *Eidolon helvum* | n14GB-B337 | Bat | Gabon |
| | **MG602640** | *Epomops franqueti* | n14GB-B280 | Bat | Gabon |
| | **MG602639** | *Epomops franqueti* | n14GB-B274 | Bat | Gabon |
| | **MG602635** | *Epomops franqueti* | n14GB-B251 | Bat | Gabon |
| | MG602644 | *Epomops franqueti* | n14GB-B292 | Bat | Gabon |
| | MG602638 | *Epomops franqueti* | n14GB-B259 | Bat | Gabon |
| | MG602645 | *Epomops franqueti* | n14GB-B314 | Bat | Gabon |
| | MG602633 | *Epomops franqueti* | n14GB-B258 | Bat | Gabon |
| | MG602641 | *Epomops franqueti* | n14GB-B281 | Bat | Gabon |
| | MG602631 | *Epomops franqueti* | n14GB-B249 | Bat | Gabon |
| | MG602642 | *Epomops franqueti* | n14GB-B282 | Bat | Gabon |
| | MG602634 | *Epomops franqueti* | n14GB-B257 | Bat | Gabon |
| | MG602637 | *Epomops franqueti* | n14GB-B245 | Bat | Gabon |
| | MG602652 | *Epomops franqueti* | n14GB-B321 | Bat | Gabon |
| | MG602648 | *Epomops franqueti* | n14GB-B335 | Bat | Gabon |
| | MG602647 | *Epomops franqueti* | n14GB-B326 | Bat | Gabon |
| | MG602646 | *Epomops franqueti* | n14GB-B319 | Bat | Gabon |
| | MG602632 | *Epomops franqueti* | n14GB-B244 | Bat | Gabon |
| | MG602643 | *Epomops franqueti* | n14GB-B289 | Bat | Gabon |
| | MG602651 | *Epomops franqueti* | n14GB-B310 | Bat | Gabon |
| | MG602653 | *Epomops franqueti* | n14GB-B303 | Bat | Gabon |
| | MG602636 | *Epomops franqueti* | n14GB-B253 | Bat | Gabon |
| | JX090243 | *Callosciurus notatus* | K12-B4 | Rodent | Malasia |

| | | | | | |
|---|---|---|---|---|---|
| *Hepatocystis* sp. | JX090244 | *Callosciurus notatus* | K12-B3 | Rodent | Malasia |
| | JX090237 | *Callosciurus notatus* | K2-A4 | Rodent | Malasia |
| | JX090235 | *Callosciurus notatus* | K6-A2 | Rodent | Malasia |
| | GU930067 | *Macaca sp.* | HB142 | Monkyes | Thailand |
| | GU929951 | *Macaca sp.* | M140_b | Monkyes | Thailand |
| | GU929975 | *Macaca sp.* | HB422 | Monkyes | Thailand |
| | EU400409 | *Macaca sp.* | MFRC11 | Monkyes | Thailand |
| | EU400408 | *Macaca sp.* | MFRC11 | Monkyes | Thailand |
| | JQ070933 | *Cercopithecus nictitans* | S3722 | Monkyes | Cameroon |
| | JQ070951 | *Cercopithecus nictitans* | 158 | Monkyes | Cameroon |
| | JQ070916 | *Cercopithecus nictitans* | 227 | Monkyes | Cameroon |
| | JQ070920 | *Cercopithecus nictitans* | S3714 | Monkyes | Cameroon |
| | JQ070914 | *Cercopithecus nictitans* | S1039 | Monkyes | Cameroon |
| | JQ070929 | *Cercopithecus nictitans* | S1089 | Monkyes | Cameroon |
| | KC262848 | - | RC5406 | Monkeys | Uganda |
| | KC262865 | - | RT1110 | Monkeys | Uganda |
| | KC262866 | - | RT1210 | Monkeys | Uganda |
| | KC262846 | - | RC4906 | Monkeys | Uganda |
| | KC262809 | - | BAB1810 | Monkeys | Uganda |
| | AF069626 | *Papio nubensis* | - | Monkeys | Ethiopia |
| | JF923760 | *Cercopithecus cephus* | WN1859/cc | Monkeys | *Gabon* |
| | JF923759 | *Mandrillus sphinx* | OLKM/msN | Monkeys | Gabon |
| | JF923757 | *Miopithecus talapoin* | WN1743/mt | Monkeys | Gabon |
| | JF923758 | *Cercopithecus cephus* | NGL/ccM | Monkeys | Gabon |
| | **MG602662** | *Cercopithecus cephus* | n14GB-PNH5 | Monkeys | Gabon |
| | **MG602663** | *Cercopithecus cephus* | n14GB-PNH3 | Monkeys | Gabon |
| | **MG602659** | *Cercocebus torquatus* | n14GB-PNH27 | Monkeys | Gabon |
| | **MG602660** | *Cercocebus torquatus* | n14GB-PNH15 | Monkeys | Gabon |
| | **MG602658** | *Mandrillus sphinx* | n14GB-PNH30 | Monkeys | Gabon |
| | **MG602657** | *Cercopithecus cephus* | n14GB-PNH9 | Monkeys | Gabon |
| | **MG602661** | *Mandrillus sphinx* | n14GB-PNH1 | Monkeys | Gabon |
| *Nycteria* sp. | KP053763 | *Rhinolophus hildebrandti* | 967 | Bat | Kenya |
| *Nycteria* sp | KF159690 | *Rhinolophus landeri* | R_lan_G3_1 | Bat | Guinea |

| | | | | | |
|---|---|---|---|---|---|
| *Nycteria gabonensis* | KX090647 | *Rhinolophus alcyone* | 289VI | Bat | DRC |
| *Polychromophilus* sp. | KF159714 | *Pipistrellus aff. grandidieri* | Pip_gran_G3_1 | Bat | Guinea |
| *Polychromophilus melanipherus* | KU182368 | *Penicillidia fulvida* | 12E233B | Bat | Gabon |
| *Plasmodium reichenowi* | GU045315 | *Pan troglodytes* | BQ642 | Apes | Gabon |
| *Plasmodium praefalciparum* | JF923761 | *Gorilla gorilla* | MOEB | Apes | Gabon |
| *Plasmodium billcollinsi* | GU815511 | *Pan troglodytes* | Atra | Apes | Ivory Coast |
| *Plasmodium blacklocki* | HM235074 | *Gorilla gorilla* | MMgor1542 | Apes | Cameroon |
| *Plasmodium gaboni* | KT824296 | *Pan troglodytes* | KApts1703_120.5 | Apes | DRC |
| *Plasmodium adleri* | GU045322 | *Gorilla gorilla* | BQ638 | Apes | Gabon |
| *Plasmodium ovale* | AF069625 | *Human* | Harding | primate | - |
| *Plasmodium ovale* | AB354571 | *Human* | - | primate | -* |
| *Plasmodium sp._DAJ-2004* | JF923753 | *Mandrillus sphinx* | Bak/ms1 | Monkeys | Gabon |
| *Plasmodium sp._DAJ-2004* | JF923755 | *Mandrillus sphinx* | MOLB | Monkeys | Gabon |
| *Plasmodium sp._DAJ-2004* | **MG602666** | *Mandrillus sphinx* | n14GB-PNH42 | Monkeys | Gabon |
| *Plasmodium sp._DAJ-2004* | **MG602665** | *Mandrillus sphinx* | n14GB-PNH54 | Monkeys | Gabon |
| *Plasmodium gonderi* | JF923751 | *Mandrillus sphinx* | Bak/ms2 | Monkeys | Gabon |
| *Plasmodium gonderi* | **MG602664** | *Mandrillus sphinx* | n14GB-PNH19 | Monkeys | Gabon |
| *Plasmodium gonderi* | **MG602656** | *Mandrillus sphinx* | n14GB-PNH33 | Monkeys | Gabon |
| *Plasmodium gonderi* | **MG602655** | *Mandrillus sphinx* | n14GB-PNH48 | Monkeys | Gabon |
| *Plasmodium gonderi* | **MG602654** | *Mandrillus sphinx* | n14GB-PNH62 | Monkeys | Gabon |
| *Plasmodium simiovale* | AF069614 | *Old world monkeys* | - | Monkeys | Sri Lanka |
| *Plasmodium inui* | EU400399 | *Macaques* | WPN4 | Monkeys | Thaïland |
| *Plasmodium vivax* | JX444723 | *Chimpanzee* | B' | Monkeys | Gabon |
| *Plasmodium vinckei* | DQ414654 | | 408XZ | Rodent | DRC |
| *Plasmodium yoelii* | AY099051 | | | | |
| *Plasmodium cyclopsi* | KF159710 | *Hipposideros cyclops* | Hip_cy_L1_1 | Bat | Liberia |
| *Plasmodium berghei* | DQ414645 | *Grammomys surdaster* | ANKA | Rodent | DRC |
| *Plasmodium voltaicum* | KF159671 | *Myonycteris angolensis* | M_ang_G1_1 | Bat | Guinea |
| *Plasmodium chabaudi* | DQ414649 | *Thamnomys rutilans* | AS | Rodent | CAR |
